# Supplementary material for: Scheduled and urgent inguinal hernia repair in Ontario, Canada between 2010 and 2022: Population-based cross sectional analysis of trends and outcomes
Source: PLoS One. 2023 Dec 22;18(12):e0296258. doi: 10.1371/journal.pone.0296258 (PMC10745156; doi:10.1371/journal.pone.0296258)
Supplement: S1 File — (DOCX) [file pone.0296258.s001.docx]

# Table S1: Administrative codes

| **CCI Code** | **Approach** | **Mesh** | **Description** |
| --- | --- | --- | --- |
| 1SY80DA | Laparoscopic |  | Repair, muscles of the chest and abdomen endoscopic [laparoscopic] approach without tissue [e.g. suturing or stapling] |
| 1SY80DAXXA | Laparoscopic |  | Repair, muscles of the chest and abdomen endoscopic [laparoscopic] approach using autograft [e.g. fascia, skin] |
| 1SY80DAXXF | Laparoscopic |  | Repair, muscles of the chest and abdomen endoscopic [laparoscopic] approach using free flap [e.g. free myocutaneous flap] |
|  |  |  |  |
| 1SY80DAXXG | Laparoscopic |  | Repair, muscles of the chest and abdomen endoscopic [laparoscopic] approach using pedicled flap [e.g. abdominis rectus or deltopectoral] |
| 1SY80DAXXL | Laparoscopic |  | Repair, muscles of the chest and abdomen endoscopic [laparoscopic] approach using xenograft [e.g. Surgis, small intestine susbmucosa] |
| 1SY80DAXXN | Laparoscopic | Yes | Repair, muscles of the chest and abdomen endoscopic [laparoscopic] approach using synthetic tissue [e.g. mesh, sponge] |
| 1SY80GDXXN | Laparoscopic | Yes | Repair, muscles of the chest and abdomen endoscopic [laparoscopic] approach with special incisional technique using synthetic tissue [e.g. mesh, sponge] |
|  |  |  |  |
| 1SY80LA | Open |  | Repair, muscles of the chest and abdomen open approach without tissue [e.g. suturing or stapling] |
| 1SY80LAFF | Open |  | Repair, muscles of the chest and abdomen open approach and using temporary abdominal closure device |
| 1SY80LAXXA | Open |  | Repair, muscles of the chest and abdomen open approach using autograft [e.g. fascia, skin] |
| 1SY80LAXXF | Open |  | Repair, muscles of the chest and abdomen open approach using free flap [e.g. free myocutaneous flap] |
|  |  |  |  |
| 1SY80LAXXG | Open |  | Repair, muscles of the chest and abdomen open approach using pedicled flap [e.g. abdominis rectus or deltopectoral] |
| 1SY80LAXXK | Open |  | Repair, muscles of the chest and abdomen open approach using homograft [e.g. tissue from deceased donor] |
| 1SY80LAXXL | Open |  | Repair, muscles of the chest and abdomen open approach using xenograft [e.g. Surgis, small intestine susbmucosa] |
| 1SY80LAXXN | Open | Yes | Repair, muscles of the chest and abdomen open approach using synthetic tissue [e.g. mesh, sponge] |
|  |  |  |  |
| 1SY80LAXXQ | Open | Yes | Repair, muscles of the chest and abdomen open approach and combined sources of tissue (e.g. mesh with autograft) |
| 1SY80LATZ | Open |  | Repair, muscles of the chest and abdomen open approach using zipper [temporary] (for repeat access to abdomen) |
| 1SY80WJ | Open |  | Repair, muscles of the chest and abdomen open approach using special excision technique |
| **Table S1. Administrative codes.** Canadian Classification of Health Interventions (CCI) codes used to identify hernia repairs and classify repairs as laparoscopic/open and mesh/no mesh.  ^a^ excluding codes involving tele-manipulation (1AY80PN, 1SY80PNXXN)  CCI – Canadian Classification of Health Interventions | | | |

# Table S2: Urgent inguinal hernia patient demographics

| **Characteristic** | **Descriptive statistic** |
| --- | --- |
|  | **7,975 (3%)** |
| Age (years), mean | 68.3 (SD 17.3) |
|  |  |
| Sex |  |
| Male | 6539 (82%) |
| Female | 1436 (18%) |
|  |  |
| American Society of Anesthesiologists score |  |
| I-II | 2257 (28%) |
| III | 3410 (43%) |
| IV | 2243 (28%) |
| V | 65 (1%) |
|  |  |
| Obesity |  |
| No | 7820 (98%) |
| Yes | 155 (1.9%) |
|  |  |
| Comorbidity score |  |
| 0 | 4747 (60%) |
| 1 | 1672 (21%) |
| 2 | 843 (11%) |
| 3+ | 713 (9%) |
|  |  |
| Travel distance (km), median IQR | 6.7 (3.1, 17.9) |
|  |  |
| Time since diagnosis (months) | 0.0 (0.0, 1.7) |
| Hospital length of stay (hours) | 51.9 (26.6, 103.7) |
|  |  |
| Type of repair |  |
| Laparoscopic | 1144 (14%) |
| Open (other technique) | 6766 (85%) |
| Open (special technique) | 65 (1%) |
|  |  |
| Use of mesh |  |
| No mesh | 1781 (22%) |
| Mesh | 6194 (78%) |
|  |  |
| Recurrent repair | 15409 (8.4%) |
|  |  |
| Rurality |  |
| Urban | 7026 (89%) |
| Rural | 899 (11%) |
|  |  |
| Deprivation |  |
| 1 (least marginalized) | 1500 (19%) |
| 2 | 1498 (19%) |
| 3 | 1522 (19%) |
| 4 | 1588 (20%) |
| 5 (most marginalized) | 1730 (22%) |
|  |  |
| Ethnic diversity |  |
| 1 (least diverse) | 1605 (20%) |
| 2 | 1561 (20%) |
| 3 | 1541 (20%) |
| 4 | 1558 (20%) |
| 5 (most diverse) | 1573 (20%) |
|  |  |
| Year |  |
| 2010 | 501 (2%) |
| 2011 | 572 (3%) |
| 2012 | 568 (3%) |
| 2013 | 611 (3%) |
| 2014 | 560 (3%) |
| 2015 | 620 (3%) |
| 2016 | 636 (3%) |
| 2017 | 617 (3%) |
| 2018 | 587 (3%) |
| 2019 | 649 (3%) |
| 2020 | 639 (4%) |
| 2021 | 693 (4%) |
| 2022 | 722 (4%) |
|  |  |
| Weekday |  |
| Sunday | 1009 (13%) |
| Monday | 1264 (16%) |
| Tuesday | 1163 (15%) |
| Wednesday | 1141 (14%) |
| Thursday | 1190 (15%) |
| Friday | 1207 (15%) |
| Saturday | 1001 (13%) |
| **Table S2: Urgent inguinal hernia patient demographics.** Descriptive statistics of patients receiving urgent inguinal hernia repair as an inpatient (public hospital) | |

# Table S3: Inguinal hernia patient demographics by era

|  | **Inpatient (Shouldice Hospital)** | | | | **Outpatient (public hospital)** | | | |
| --- | --- | --- | --- | --- | --- | --- | --- | --- |
|  | **Pre-COVID-19 era** | **COVID-19 era** | **OR (95% CI)^a^** | **Pre-COVID-19 era** | | **COVID-19 era** | **OR (95% CI)^a^** |  |
|  | **N=11,509** | **N=10,972** |  | **N=42,517** | | **N=34,657** |  |  |
| Age (years)^b^ | 57.8 (SD 14.3) | 59.6 (SD 14.1) | 1.03 (1.01-1.05) | 60.7 (SD 15.2) | | 61.9 (SD 15.2) | 1.03 (1.02-1.04) |  |
|  |  |  |  |  | |  |  |  |
| Sex |  |  |  |  | |  |  |  |
| Male | 11014 (96%) | 10447 (95%) | 1.0 (ref) | 39092 (92%) | | 32035 (92%) | 1.0 (ref) |  |
| Female | 495 (4.3%) | 525 (4.8%) | 1.08 (0.94-1.23) | 3425 (8.1%) | | 2621 (7.6%) | 0.93 (0.88-0.99) |  |
|  |  |  |  |  | |  |  |  |
| ASA score |  |  |  |  | |  |  |  |
| I-II | 10724 (93%) | 9288 (85%) | 1.0 (ref) | 24306 (57%) | | 17586 (51%) | 1.0 (ref) |  |
| III | 771 (7%) | 1646 (15%) | 2.13 (1.93-2.35) | 15748 (37%) | | 14334 (41%) | 1.24 (1.20-1.29) |  |
| IV-V | 14 (<1%) | 38 (<1%) | 2.17 (1.15-4.07) | 2463 (6%) | | 2736 (8%) | 1.55 (1.45-1.66) |  |
|  |  |  |  |  | |  |  |  |
| Obesity |  |  |  |  | |  |  |  |
| No | 11509 (100%) | 10972 (100%) | n/a | 42026 (99%) | | 34111 (98%) | 1.0 (ref) |  |
| Yes | 0 (0%) | 0 (0%) | n/a | 491 (1.2%) | | 545 (1.6%) | 1.22 (1.07-1.39) |  |
|  |  |  |  |  | |  |  |  |
| Comorbidity score |  |  |  |  | |  |  |  |
| 0 | 10140 (88%) | 9032 (82%) | 1.0 (ref) | 34930 (82%) | | 28194 (81%) | 1.0 (ref) |  |
| 1 | 1198 (10%) | 1650 (15%) | 1.26 (1.15-1.37) | 5614 (13%) | | 4830 (14%) | 0.96 (0.91-1.00) |  |
| 2 | 136 (1.2%) | 235 (2.1%) | 1.34 (1.09-1.63) | 1262 (3.0%) | | 1057 (3.1%) | 0.88 (0.80-0.96) |  |
| 3+ | 35 (0.3%) | 55 (0.5%) | NR | 711 (1.7%) | | 574 (1.7%) | 0.81 (0.72-0.91) |  |
|  |  |  |  |  | |  |  |  |
| Travel distance (km)^b^ | 64.5 (19.3, 156.3) | 60.4 (19.6, 141.4) | 1.00 (0.99-1.01) | 8.9 (3.0, 23.3) | | 9.1 (3.9, 23.2) | 1.00 (1.00-1.02) |  |
| Time until repair (mos)^b^ | 2.6 (1.6, 4.2) | 3.9 (2.3, 6.1) | 1.16 (1.14-1.17) | 2.7 (1.4, 5.0) | | 3.9 (1.8, 7.0) | 1.12 (1.11-1.12) |  |
| Laparoscopic approach | 0 (0%) | 0 (0%) | n/a | 8044 (23%) | | 9056 (21%) | 1.11 (1.07-1.15) |  |
| Mesh | 23 (0.2%) | 64 (0.6%) | 2.94 (1.75-4.95) | 38936 (92%) | | 32510 (94%) | 1.35 (1.28-1.44) |  |
| Repeat repair | 577 (5.0%) | 510 (4.7%) | 0.83 (0.73-0.95) | 3757 (8.8%) | | 2848 (8.2%) | 0.85 (0.81-0.90) |  |
|  |  |  |  |  | |  |  |  |
| Rurality |  |  |  |  | |  |  |  |
| Urban | 10227 (89%) | 9761 (89%) | 1.0 (ref) | 36017 (85%) | | 28994 (84%) | 1.0 (ref) |  |
| Rural | 1211 (11%) | 1156 (11%) | 1.04 (0.94-1.16) | 6218 (15%) | | 5419 (16%) | 1.10 (1.05-1.15) |  |
|  |  |  |  |  | |  |  |  |
| Deprivation |  |  |  |  | |  |  |  |
| 1 (least deprived) | 3504 (31%) | 3459 (31%) | 1.0 (ref) | 9430 (23%) | | 8125 (24%) | 1.0 (ref) |  |
| 2 | 2772 (24%) | 2765 (25%) | 1.02 (0.94-1.09) | 8917 (21%) | | 7403 922%) | 0.96 (0.92-1.00) |  |
| 3 | 2141 (19%) | 2011 (19%) | 0.96 (0.89-1.05) | 8265 (20%) | | 6564 (19%) | 0.92 (0.88-0.97) |  |
| 4 | 1736 (15%) | 1579 (15%) | 0.91 (0.83-0.99) | 7822 (19%) | | 6309 (19%) | 0.93 (0.89-0.97) |  |
| 5 (most deprived) | 1192 (11%) | 1032 (10%) | 0.87 (0.79-0.97) | 7280 (18%) | | 5703 (17%) | 0.91 (0.86-0.95) |  |
|  |  |  |  |  | |  |  |  |
| Ethnic diversity |  |  |  |  | |  |  |  |
| 1 (least diverse) | 2092 (18%) | 1904 (18%) | 1.0 (ref) | 3.9 (1.8, 7.0) | | 8159 (24%) | 1.0 (ref) |  |
| 2 | 2126 (19%) | 2013 (19%) | 1.03 (0.94-1.13) | 8528 (20%) | | 7192 921%) | 0.97 (0.92-1.01) |  |
| 3 | 2508 (22%) | 2471 (23%) | 1.08 (0.98-1.19) | 7915 (19%) | | 6364 (19%) | 0.92 (0.87-0.96) |  |
| 4 | 2582 (23%) | 2460 (23%) | 1.05 (0.95-1.15) | 7640 (18%) | | 6105 (18%) | 0.92 (0.87-0.96) |  |
| 5 (most diverse) | 2037 (18%) | 1998 (18%) | 1.10 (0.99-1.22) | 8205 (20%) | | 6284 (18%) | 0.93 (0.88-0.98) |  |
| **Table S3: Inguinal hernia patient demographics by era**. Sociodemographics and comparison of patients receiving inguinal hernia repair in the pre-COVID-19 era (March 2017 through December 2019) compared with the COVID-19 era (March 2020 through December 2022). Results are reported separately for repairs performed at Shouldice Hospital and the public hospital outpatient settings.  Pre-COVID-19 era was March 2017 through December 2019; COVID-19 era was March 2020 through December 2022  ^a^ odds ratios (OR) and 95% confidence intervals (CI) were adjusted for all variables shown  ^b^ odds ratios for continuous variables represent a 10-year increase in age, a 50-km increase in travel distance, and a 1-month increase in the time until repair | | | | | | | | |

# Table S4: Outcomes for inguinal repair: descriptive statistics

|  | Wait-time (days)^a^ | 90-day readmissions | 1-year re-operations^b^ |
| --- | --- | --- | --- |
|  | Median (IQR) | N (%) | N (%) |
| Urgency |  |  |  |
| Urgent | 0 (0, 51) | 973 (12.5%) | 289 (4.0%) |
| Scheduled | 83 (43, 155) | 6471 (2.6%) | 4116 (1.8%) |
|  |  |  |  |
| Scheduled over time |  |  |  |
| 2010 | n/a | 571 (2.9%) | 357 (1.8%) |
| 2011 | 72 (42, 132) | 523 (2.6%) | 375 (1.9%) |
| 2012 | 74 (40, 138) | 537 (2.7%) | 347 (1.7%) |
| 2013 | 73 (38, 134) | 502 (2.5%) | 383 (1.9%) |
| 2014 | 73 (37, 134) | 536 (2.7%) | 375 (1.9%) |
| 2015 | 73 (40, 139) | 521 (2.55) | 332 (1.6%) |
| 2016 | 72 (37, 139) | 541 (2.7%) | 385 (1.9%) |
| 2017 | 77 (41, 142) | 502 (2.5%) | 370 (1.8%) |
| 2018 | 78 (43, 146) | 519 (2.6%) | 394 (2.0%) |
| 2019 | 90 (48, 156) | 527 (2.7%) | 303 (1.6%) |
| 2020 | 117 (54, 212) | 376 (2.4%) | 233 (1.5%) |
| 2021 | 105 (56, 190) | 409 (2.4%) | 262 (1.5%) |
| 2022 | 133 (69, 213) | 406 (2.5%) | n/a |
| **Table S4: Outcomes for inguinal repair: descriptive statistics.** The median time until diagnosis and the proportion of repairs with a 90-day readmission or 1-year reoperations. Results are stratified by urgent versus scheduled repair, and year of scheduled repair.  ^a^ median (interquartile range) days from first hernia diagnostic code until repair  ^b^ restricted to primary elective (scheduled) operations occurring between January 1, 2010 and December 31, 2021  IQR – interquartile range  n/a – not available | | | |

# Fig S1: Patient selection

Inpatient hernia repairs (DAD)

January 2010-Decmeber 2023

N=141,079 records

Outpatient hernia repairs (NACRS)

January 2010-Decmeber 2023

N=315,889 records

- Exclude on same day as DAD record (n=163)
- Only keep K40-K43 (exclude 6424)
- Only keep K40-K43 (exclude 7590)

N=442,791 records

Exclude

- Exclude duplicates (n=14)
- Exclude tele-manipulation (n=9)

N=442,768 records

N=404,403 unique patients

Exclude

- Age <18 years (n=22,496)
- Exclude ventral hernia (n=78,798), umbilical (n=82,418), and femoral (n=7,061) repairs

N=342,429 records

N=318,412 unique patients

# Fig S2: Scheduled inguinal hernia repair by type and setting

A) Number of repairs


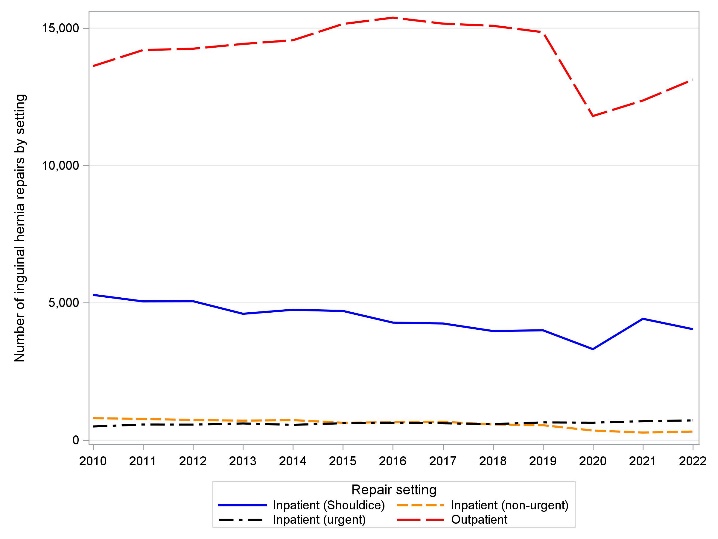


B) Percent of repairs


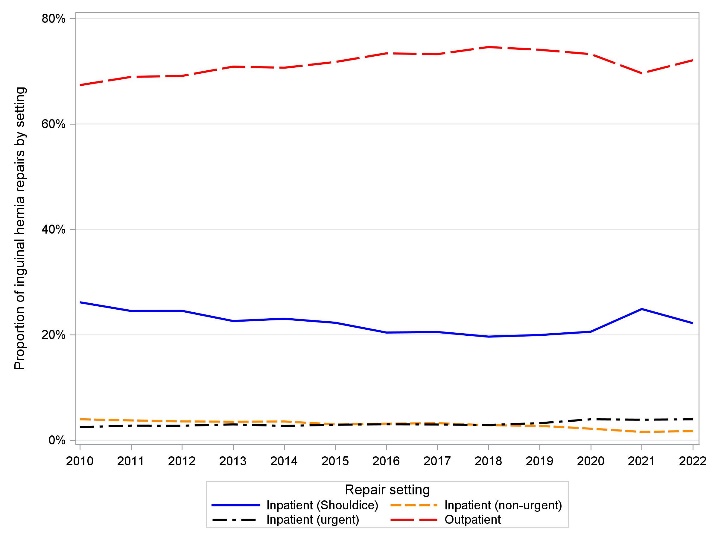


**S2 Fig. Annual scheduled inguinal hernia repair by setting.** Number (A) and proportion (B) of inguinal hernia repairs performed in the outpatient public setting, inpatient public setting (scheduled), inpatient public setting (urgent), and inpatient Shouldice Hospital setting

# Fig S3: Hernia repair approach by type

A) Laparoscopic repair for scheduled repairs


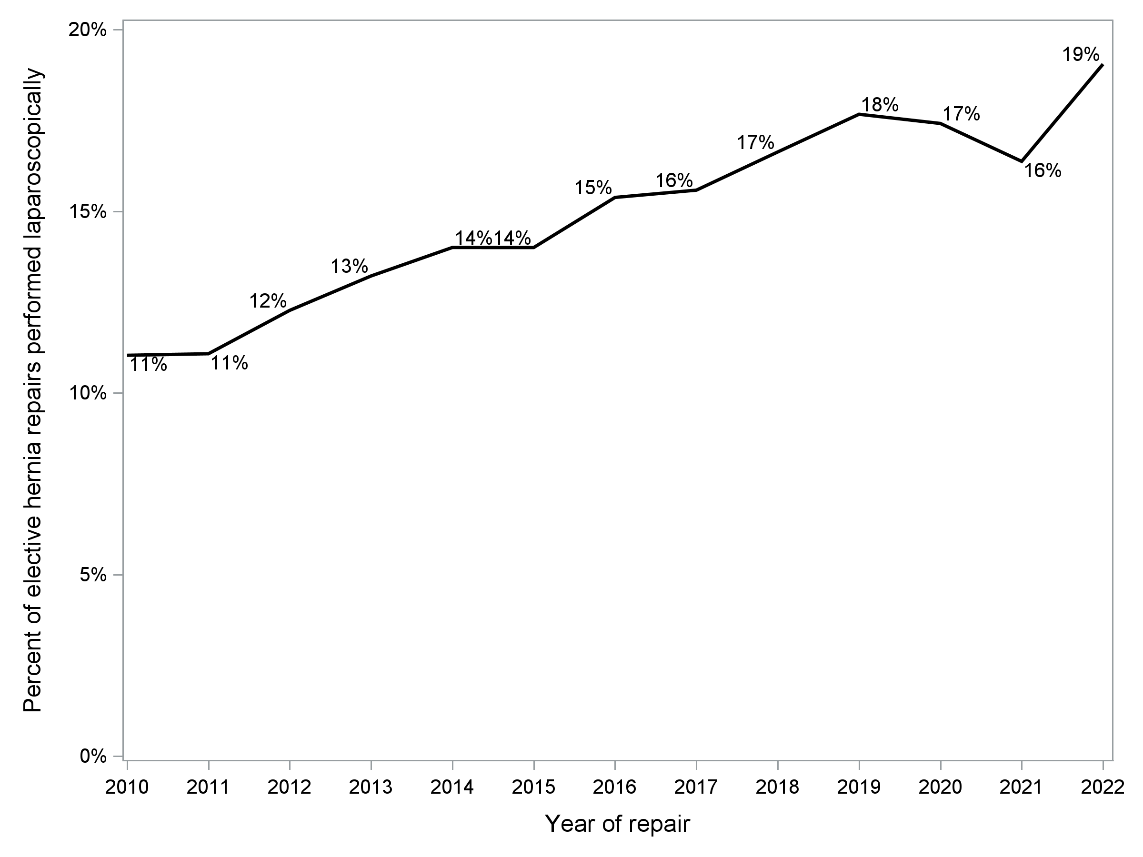


B) Use of mesh


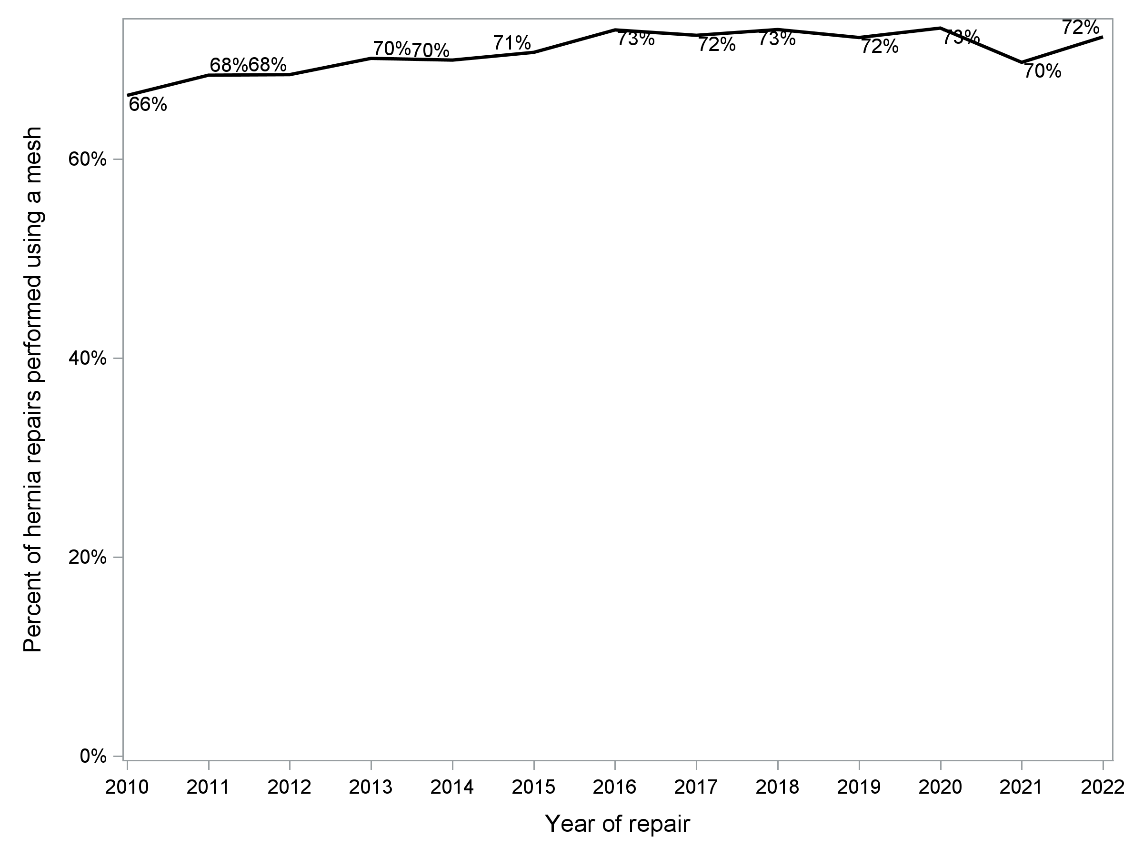


**S3 Fig. Inguinal hernia repair by approach.** Proportion of inguinal hernias performed using the laparoscopic approach (A) or use of mesh (B) by year of repair.

# Fig S4: Total hospital length of stay by setting
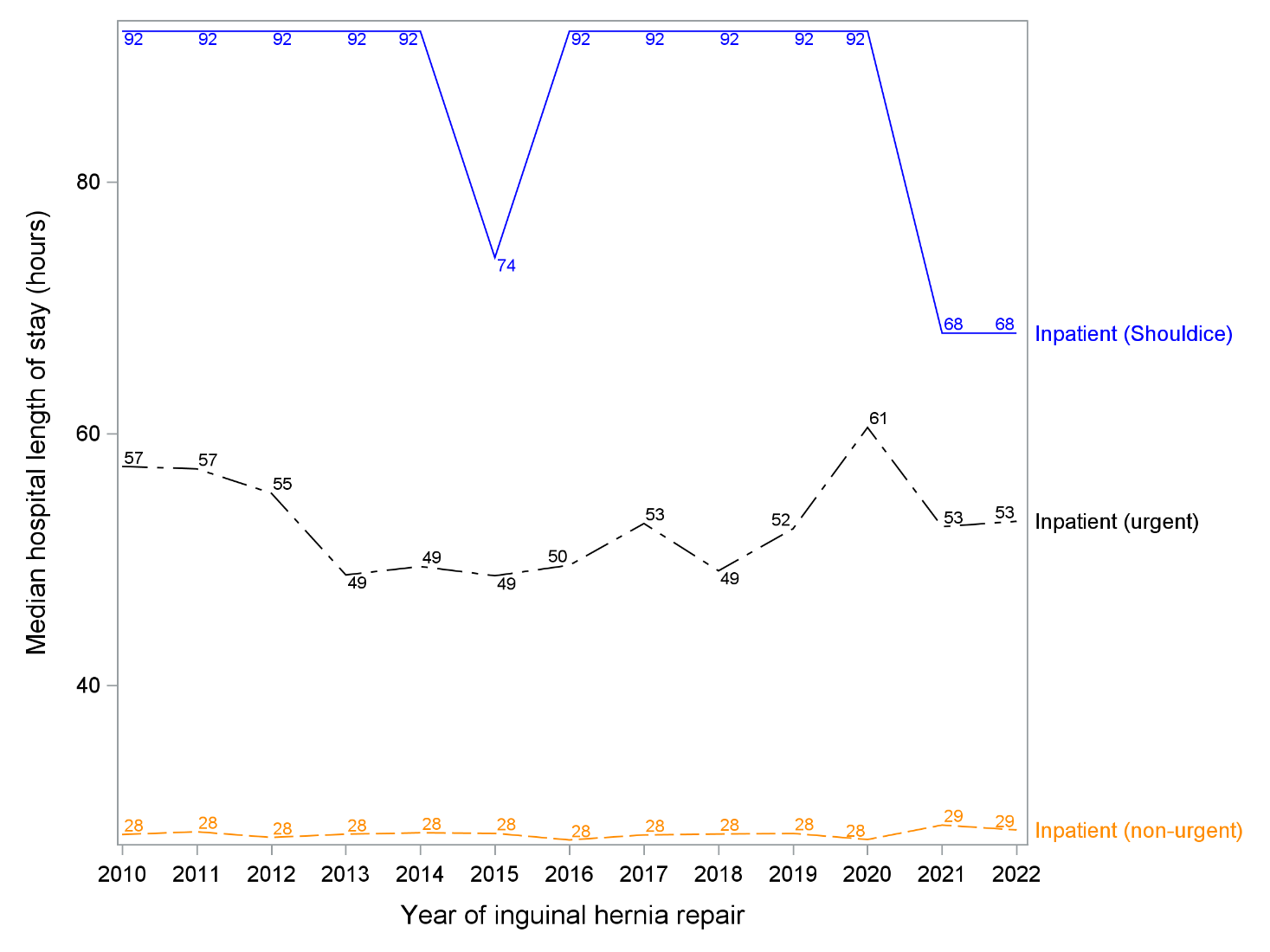


**S4 Fig. Total hospital length of stay by setting.** Median total hospital length of stay by year of hernia repair by setting for inguinal hernia repairs.
