# Supplementary material for: Scheduled and urgent inguinal hernia repair in Ontario, Canada between 2010 and 2022: Population-based cross sectional analysis of trends and outcomes
Source: PLoS One. 2023 Dec 22;18(12):e0296258. doi: 10.1371/journal.pone.0296258 (PMC10745156; doi:10.1371/journal.pone.0296258)
Supplement: Attachment — Submitted filename: Response to reviewers - hernia repair - PONE-D-23-25270_R1.docx [file pone.0296258.s003.docx]

**November 30, 2023**

**PONE-D-23-25270R2**

Dear Dr Sikakulva,

On behalf of the co-authors, we thank you and the reviewers for the review, and we appreciate the rather quick turn-around on this. We provide point-by-point responses below and for convenience, we keep previous responses and letters below.

Thank you for your time and consideration

Sincerely,

Steven Habbous, PhD

Epidemiologist

Ontario Health (Strategic Analytics)

Email: [steven.habbous@ontariohealth.on.ca](mailto:steven.habbous@ontariohealth.on.ca)

**November 27, 2023**

**PONE-D-23-25270R1**

Scheduled and urgent inguinal hernia repair in Ontario, Canada between 2010 and 2022: population-based cross sectional analysis of trends and outcomes

PLOS ONE

Dear Dr. Habbous,

Thank you for submitting your manuscript to PLOS ONE. After careful consideration, we feel that it has merit but does not fully meet PLOS ONE’s publication criteria as it currently stands. Therefore, we invite you to submit a revised version of the manuscript that addresses the points raised during the review process.

Please submit your revised manuscript by Jan 11 2024 11:59PM. If you will need more time than this to complete your revisions, please reply to this message or contact the journal office at [plosone@plos.org](mailto:plosone@plos.org). When you're ready to submit your revision, log on to <https://www.editorialmanager.com/pone/> and select the 'Submissions Needing Revision' folder to locate your manuscript file.

Please include the following items when submitting your revised manuscript:

- A rebuttal letter that responds to each point raised by the academic editor and reviewer(s). You should upload this letter as a separate file labeled 'Response to Reviewers'.
- A marked-up copy of your manuscript that highlights changes made to the original version. You should upload this as a separate file labeled 'Revised Manuscript with Track Changes'.
- An unmarked version of your revised paper without tracked changes. You should upload this as a separate file labeled 'Manuscript'.

If you would like to make changes to your financial disclosure, please include your updated statement in your cover letter. Guidelines for resubmitting your figure files are available below the reviewer comments at the end of this letter.

If applicable, we recommend that you deposit your laboratory protocols in protocols.io to enhance the reproducibility of your results. Protocols.io assigns your protocol its own identifier (DOI) so that it can be cited independently in the future. For instructions see: [https://journals.plos.org/plosone/s/submission-guidelines#loc-laboratory-protocols](https://can01.safelinks.protection.outlook.com/?url=https%3A%2F%2Fjournals.plos.org%2Fplosone%2Fs%2Fsubmission-guidelines%23loc-laboratory-protocols&data=05%7C01%7Csteven.habbous%40ontariohealth.ca%7Cd24a78e2c71c4faeb0e608dbf0152ebc%7C4ef96c5cd83f466ba478816a5bb4af62%7C0%7C0%7C638367746001587452%7CUnknown%7CTWFpbGZsb3d8eyJWIjoiMC4wLjAwMDAiLCJQIjoiV2luMzIiLCJBTiI6Ik1haWwiLCJXVCI6Mn0%3D%7C3000%7C%7C%7C&sdata=Eyqlp8jiNoXKKemUNTr62lflErsZ0zCEiVVn3xTImRQ%3D&reserved=0). Additionally, PLOS ONE offers an option for publishing peer-reviewed Lab Protocol articles, which describe protocols hosted on protocols.io. Read more information on sharing protocols at [https://plos.org/protocols?utm_medium=editorial-email&utm_source=authorletters&utm_campaign=protocols](https://can01.safelinks.protection.outlook.com/?url=https%3A%2F%2Fplos.org%2Fprotocols%3Futm_medium%3Deditorial-email%26utm_source%3Dauthorletters%26utm_campaign%3Dprotocols&data=05%7C01%7Csteven.habbous%40ontariohealth.ca%7Cd24a78e2c71c4faeb0e608dbf0152ebc%7C4ef96c5cd83f466ba478816a5bb4af62%7C0%7C0%7C638367746001599022%7CUnknown%7CTWFpbGZsb3d8eyJWIjoiMC4wLjAwMDAiLCJQIjoiV2luMzIiLCJBTiI6Ik1haWwiLCJXVCI6Mn0%3D%7C3000%7C%7C%7C&sdata=bfL9LwBjehuns3pDGbD4VS%2B3q3J90QTAEODunEHE8Ok%3D&reserved=0).

We look forward to receiving your revised manuscript.

Kind regards,

Franck Katembo Sikakulya
Academic Editor
PLOS ONE

Journal Requirements:

Please review your reference list to ensure that it is complete and correct. If you have cited papers that have been retracted, please include the rationale for doing so in the manuscript text, or remove these references and replace them with relevant current references. Any changes to the reference list should be mentioned in the rebuttal letter that accompanies your revised manuscript. If you need to cite a retracted article, indicate the article’s retracted status in the References list and also include a citation and full reference for the retraction notice.

**Additional Editor Comments:**

Please find attached the comments from reviewers for you to improve the draft before its consideration.

[Note: HTML markup is below. Please do not edit.]

**Reviewers' comments:**

**REVIEWER #1:**

This paper is much improved, and I now have only a few minor issues:

**Comment #1:** In the Abstract please put in 15% after '... 8,162 {15%) fewer ....'

**Response:** Thank you for the suggestion, we moved the percent to after the word “fewer”.

**Comment #2:** Although the setting is mentioned in the Results, I think it should also have a separate heading in the Methods - e.g. Setting - patients management in three clinical setting was examined: Public hospital in-patient care, Private hospital in-patient care, and Public hospital out-patient care

**Response:** This is a good suggestion to improve the clarity of the methods. In the abstract, we added the following sentence: “Patients managed in three clinical settings were examined: public hospital in-patient, private hospital in-patient (Shouldice Hospital), and public hospital out-patient.” In the manuscript, we split out the “Covariates” subsection of the methods into “Setting” [describes patient groupings into public inpatient, private inpatient, public outpatient] and “Covariates [describes the other covariates].

**Comment #3:** The Results are presented rather unusually. Most papers start the Results with patient demographics usually presented in Table, followed by the rest of the Results. This paper does not do that, but starts with Table 2, and then introduces the demographics etc later on, with Table 1 following Table 2. This is most confusing. I realise there is a lot in Table 1. Therefore it might be better to split the demographics off as a separate table. However, there is no reason not to refer to Table 1, then Table 2, and then go back to Table 1 is needed.

**Response:** Thank you for noticing this. Indeed we previously had Table 2 listed as Table 1 (for the reason you describe), but ended up moving it to align with the presentation of the results. We added a new Table 1 (“Cohort characteristics”), which is a simplified (e.g. aggregated) descriptive statistics table the most pertinent cohort factors.

**REVIEWER #2:**

**Comment #3:** I would like to thank the authors for their efforts to revise the manuscript per reviewer's comments.

**Response:** Thank you your help improving this manuscript.

**November 6, 2023**

**PONE-D-23-25270**

Dear Dr Sikakulva,

On behalf of the co-authors, we thank you and the reviewers for the review and suggestions to improve the manuscript. We provide point-by-point responses below. No changes were made to our conflicts of interest or the financial discloser, and for convenience, the original cover letter is included below. Regarding access to data, we are not permitted to share data publicly for legal and privacy reasons (as per our privacy office), so I believe our data availability statement is appropriate (non-author point of contact [Datarequest@ontariohealth.ca](mailto:Datarequest@ontariohealth.ca)). A copy of our privacy assessment was uploaded as “Other” in the initial submission.

Thank you for your time and consideration

Sincerely,

Steven Habbous, PhD

Epidemiologist

Ontario Health (Strategic Analytics)

Email: [steven.habbous@ontariohealth.on.ca](mailto:steven.habbous@ontariohealth.on.ca)

August 2, 2023

Re: Inguinal, umbilical, and femoral hernia repairs

Dear editor,

On behalf of our co-authors, we would like to submit our original article entitled “**Scheduled and urgent inguinal, umbilical, and femoral hernia repair in Ontario, Canada between 2010 and 2022: population-based cross sectional analysis of trends and outcomes**” for consideration in your journal.

**Brief overview:** The COVID-19 pandemic has resulted in a marked reduction in scheduled hernia repair surgeries. As one of the most commonly performed procedures, we estimate 14,070 fewer inguinal, umbilical, and femoral hernia repairs were performed than expected during the pandemic based on pre-pandemic trends. One strategy to increase the number of surgeries is to transition more procedures to the outpatient setting. In Ontario’s public payer health care system, we found that the proportion of scheduled repairs performed in public hospitals that were in the outpatient setting exceeded 95%. However, as Shouldice Hospital performs a quarter of all hernia repairs in the province—and delivers these exclusively as inpatient cases—there is a sizable impact of the facility on Ontario’s overall provincial outpatient rate. During the pandemic, length-of-stay at Shouldice Hospital was lower by an average 24h and the time until scheduled surgery increased in all settings, but patient outcomes (90-day readmissions and 1-year reoperations) were not worse.

**Data availability:** Ontario Health is prohibited from making the data used in this research publicly accessible if it includes potentially identifiable personal health information and/or personal information as defined in Ontario law, specifically the Personal Health Information Protection Act (PHIPA) and the Freedom of Information and Protection of Privacy Act (FIPPA). Due to these legal and ethical restrictions, data will not be made publicly available. However, upon request, data de-identified to a level suitable for public release may be provided.

**Billing:** Steven Habbous (lead and corresponding author) is affiliated with Western University, Ontario, Canada. Given the relationship between Western University and PLoS journals, the university can be billed directly if accepted for publication.

The contents of this manuscript are not currently under consideration for publication elsewhere. All authors of this research paper participated in the preparation of this manuscript and any conflicts of interest are detailed in the manuscript. On behalf of the authors, thank you in advance for your time and consideration.

Sincerely,

Steven Habbous, PhD

Epidemiologist

Ontario Health (Strategic Analytics)

Email: [steven.habbous@ontariohealth.on.ca](mailto:steven.habbous@ontariohealth.on.ca)

**EDITOR COMMENTS:**

**Comment:** This paper contains important information, and therefore we strongly suggest they re-draft this paper along the lines suggested by reviewers

**Response:** Thank you for the positive note. We ultimately restricted the manuscript only to inguinal hernias as per Reviewer #1’s comments. We reorganized the results section to improve the flow of the manuscript, which we hope will help the readers.

**REVIEWER COMMENTS:**

**Reviewer: 1**
**Comment #1:** This paper reports the change in rate of hernia operations during the COVID-19 pandemic. Its major finding was that there were fewer operations performed, except in a single private hospital that performed more surgery and reduced its length of stay. Although the Abstract gives the decline in surgeries performed as numbers it does not give the percentage drop, which it should.

**Response:** Thank you for the suggestion, we added the percent reduction (15%) in the abstract

**Comment #2:** This paper is information heavy, and really is a case of information overload and displays a dogged determination to ensure the reader cannot see the wood for the trees. As the author’s concede in their Discussion, uniquely the 89 bed Shouldice Hospital that only specializes in hernia surgery performs 18.9% of all hernia surgeries in Ontario. Moreover, nearly 90% of all hernia operations performed in public hospitals are outpatient procedures. Therefore, the features that make this paper unique and of interest to international readers is the impact, if any, the COVID-19 pandemic had on these two practice innovations. The authors should re-draft their paper to make the changes in 1. in-patient public hospital hernia surgery, 2. out-patient public hospital hernia surgery, and 3. in-patient private specialist hernia surgery clearer. It took me a long time to sort all this out, and reading and re-reading the data to realize that the outcomes for all these three were NOT the same.

**Response:** Thank you for the comment and we agree that there is a lot of information presented in this manuscript. To improve the clarity of the work, we removed umbilical and femoral hernia repairs and instead focus solely on inguinal hernia. We reorganized the results as follows, examining how the COVID-19 pandemic affected:

1. Surgical volumes and population rates over time
2. Use of hospital setting (e.g. outpatient versus public-inpatient versus Shouldice versus urgent)
3. Patient characteristics by setting and over time
4. Timeliness of care (e.g. time from diagnosis until surgery)
5. The provision of care (hospital length of stay)
6. Patient outcomes (e.g. readmission and revision)

To organize the paper further, in addition to providing more meaningful subheadings, we also replaced Table 1 with Table S2 (patient demographics by setting) and explicitly list out the different settings a patient can receive care early in the methods (Covariates subsection). One of the objectives of this work was to assess the quality of care, so it was important to report on the number and rate of urgent hernia repairs because it was conceivable that reduced elective procedures would result in worsening conditions necessitating urgent repair. For completeness, we also report the patient characteristics for the urgent group for the interested reader, but we relegate this to the supplement to reduce clutter in the prose. We also moved the main outcomes table to the prose (now Table 3). It was previously left to the supplement, but now that it’s only restricted to inguinal repairs, the manuscript is less cluttered.

**Comment #3:** Hernia repair is arguably the quintessential elective surgery, as for most patients it is often only an inconvenience as strangulation and other life-threatening complications are relatively uncommon. For simplicity of presentation, it might be reasonable to combine all three, or as the risk of complications may not be the same for all of them, confining most of the analysis to inguinal hernia. The point being that what the reader wants to know are:
1. The increasing trend in out-patient surgery only declined slightly during the pandemic.
2. The declining trend in private in-patient surgery was reversed, and the length of stay shortened despite patients with more co-morbidity being treated.
3. The declining trend in public in-patient surgery continued.
4. What happened to the need for urgent repair? These data need to be qualified by / related to the duration of symptoms, the availability of private in-patient surgery, public in-patient, and out-patient surgery. My hypothesis would be that those patients who had symptoms the longest, and lived furthest from any of these available services or who could least afford to avail of them would be most likely to require urgent surgery. A logistic regression model might also consider other variables.
As I consider this paper needs a complete re-draft, I will not go through it in too much detail. However, I note that the Methods section does not mention the clinical Settings, and Table 1 is far too detailed, and it is not clear what the percentages provided are percentages of. Furthermore, the Figures have no adequate legends.

**Response:** We have indeed taken this comment to heart, and have restricted the analyses to inguinal repairs in the revision. The first paragraph of the discussion that summarizes the findings now includes these points:

“We observed that the increasing trend in outpatient surgery only declined slightly during the pandemic, while the decline in public inpatient surgery continued. The declining trend in private inpatient surgery (Shouldice Hospital) was reversed during the pandemic, and the length of stay was shortened despite treating more patients with comorbidity.”

Since we removed femoral and umbilical hernias, the former Table 1 was not considered helpful, and was replaced with what was formerly supplementary Table S2 (patient characteristics by setting; now Table 2). The urgent inpatient group was left to the supplement as mentioned above. We have added “travel distance” to the list of covariates and report these in Table 2. Interestingly, there was no meaningful difference in travel distance for patients treated at public hospitals (median 9.3km for outpatient; 8.2km for non-urgent inpatient) compared with Shouldice Hospital (median 59km). As the rate of urgent repairs remained steady over time and is largely out of the control of the healthcare system, we did not examine this subgroup further.

Note that the figure legends are embedded within the prose rather than associated with the figure as per PLoS guidelines. We also clarify the meaning of the percentages in Table 2 in the revision.

**Reviewer: 2**

Abstract
**Comment #1:** Unsure how the study is a “case-control” study. This term is not used in the methods section of the manuscript.

**Response:** Thank you for noticing this, we have removed “case-control” from the methods section of the abstract.

**Comment #2:** The methods section of the abstract could use more detail and there is room within the 300 word limit to implement this.

**Response:** Great point, we have extended the abstract to include more methodological details (a brief primer on Shouldice Hospital; regression method; covariates used for adjustment).

Introduction

**Comment #3:** Conveying the high proportion of hernia repairs in Ontario that performed at the Shouldice Hospital would help convey why this institution of is of particular interest. As some analyses are related specifically to operations performed at Shouldice it would also be helpful for the hospital to appear in the objective.

**Response:** This is a good point. We have added the following to the concluding statement of the introduction section: “We examine where patients receive surgery, contrasting those treated at public hospitals with those treated at the Shouldice Hospital”. We also add that the Shouldice Hospital performs a large percentage of all inguinal repairs in the province, highlighting its importance.

**Comment #4:** It is clear from the introduction why trends in the volume of surgical hernia repairs may have changed due to the COVID-19 pandemic but not clear why outcomes following surgery may have been impacted. I would suggest the authors provide some background into the rationale for looking for changes in outcomes.

**Response:** This is another good point. We have added the following to the concluding statement of the introduction section: “We assessed the change in patient characteristics and outcomes over time to ensure that the quality of care was not adversely affected due to hospital pressures imposed by the COVID-19 pandemic.”

Methods

**Comment #5:** There is no mention of Shouldice Hospital in the Methods section despite it having it a separate subheading in the Results section. Please describe how in the methods how Shouldice will be analyzed separately.

**Response:** Thank you for noting this. The “Shouldice” subheading in the results is no longer present. We added the following to the methods section to clearly describe the breakdown of the cohort by setting:

“The repair setting was classified as outpatient if captured by the CIHI-NACRS database and inpatient if captured by CIHI-DAD. Inpatient procedures were further subclassified as Shouldice Hospital if the procedure was performed there; non-urgent inpatient; and urgent inpatient. Urgent operations were defined as an admission with entry code = E (admitted through the emergency department), admission category = U (urgent), or if the patient arrived by ambulance.”

**Comment #6:** Could the authors add more detail on the regression model, including on how autocorrelation was considered?

**Response:** Certainly. The regression model used the weekly count as a predictor. The number of procedures performed in a given week is not expected to depend on the number of procedures performed in the previous week, thus, we did not consider autocorrelation. Instead, we expected the week number itself (1-52) to more aptly predict week-to-week variability, because this is better suited to incorporate consistent trends related to holidays and vacation times. Moreover, autocorrelation models require assumptions about the nature of the correlation. The model we used had excellent fit (very small and acceptable error), so we did not opt to search for a more complex model. Lastly, the estimated number of hernia repairs “missed” during the pandemic was calculated quite accurately using the regression model.

6. PLOS authors have the option to publish the peer review history of their article ([what does this mean?](https://can01.safelinks.protection.outlook.com/?url=https%3A%2F%2Fjournals.plos.org%2Fplosone%2Fs%2Feditorial-and-peer-review-process%23loc-peer-review-history&data=05%7C01%7Csteven.habbous%40ontariohealth.ca%7Cbe54471f52754efd03f708dbd56e4e87%7C4ef96c5cd83f466ba478816a5bb4af62%7C0%7C0%7C638338442184918838%7CUnknown%7CTWFpbGZsb3d8eyJWIjoiMC4wLjAwMDAiLCJQIjoiV2luMzIiLCJBTiI6Ik1haWwiLCJXVCI6Mn0%3D%7C3000%7C%7C%7C&sdata=nMAAo3llMVtZZxk70kk9a25lof3OkBAHpCHGh2wz3e0%3D&reserved=0)). If published, this will include your full peer review and any attached files.


If you choose “no”, your identity will remain anonymous but your review may still be made public.


**Do you want your identity to be public for this peer review?** For information about this choice, including consent withdrawal, please see our [Privacy Policy](https://can01.safelinks.protection.outlook.com/?url=https%3A%2F%2Fwww.plos.org%2Fprivacy-policy&data=05%7C01%7Csteven.habbous%40ontariohealth.ca%7Cbe54471f52754efd03f708dbd56e4e87%7C4ef96c5cd83f466ba478816a5bb4af62%7C0%7C0%7C638338442184918838%7CUnknown%7CTWFpbGZsb3d8eyJWIjoiMC4wLjAwMDAiLCJQIjoiV2luMzIiLCJBTiI6Ik1haWwiLCJXVCI6Mn0%3D%7C3000%7C%7C%7C&sdata=TVBwc2OuJdnw%2FLNSv%2BsvPpoZlh6NfpiicF8XIbnSxZk%3D&reserved=0).

Reviewer #1: **Yes:** John Kellett

Reviewer #2: No

**Oct 18, 2023**

**PONE-D-23-25270**

Scheduled and urgent inguinal, umbilical, and femoral hernia repair in Ontario, Canada between 2010 and 2022: population-based cross sectional analysis of trends and outcomes

PLOS ONE

Dear Dr. Habbous,

Thank you for submitting your manuscript to PLOS ONE. After careful consideration, we feel that it has merit but does not fully meet PLOS ONE’s publication criteria as it currently stands. Therefore, we invite you to submit a revised version of the manuscript that addresses the points raised during the review process.

Please submit your revised manuscript by Dec 02 2023 11:59PM. If you will need more time than this to complete your revisions, please reply to this message or contact the journal office at [plosone@plos.org](mailto:plosone@plos.org). When you're ready to submit your revision, log on to [https://www.editorialmanager.com/pone/](https://can01.safelinks.protection.outlook.com/?url=https%3A%2F%2Fwww.editorialmanager.com%2Fpone%2F&data=05%7C01%7Csteven.habbous%40ontariohealth.ca%7Cbe54471f52754efd03f708dbd56e4e87%7C4ef96c5cd83f466ba478816a5bb4af62%7C0%7C0%7C638338442184762483%7CUnknown%7CTWFpbGZsb3d8eyJWIjoiMC4wLjAwMDAiLCJQIjoiV2luMzIiLCJBTiI6Ik1haWwiLCJXVCI6Mn0%3D%7C3000%7C%7C%7C&sdata=iyasjGT94KTWbZWXbB%2FH7CoxKousX1mvFSXr13Q%2FkZs%3D&reserved=0) and select the 'Submissions Needing Revision' folder to locate your manuscript file.

Please include the following items when submitting your revised manuscript:

1. A rebuttal letter that responds to each point raised by the academic editor and reviewer(s). You should upload this letter as a separate file labeled 'Response to Reviewers'.
2. A marked-up copy of your manuscript that highlights changes made to the original version. You should upload this as a separate file labeled 'Revised Manuscript with Track Changes'.
3. An unmarked version of your revised paper without tracked changes. You should upload this as a separate file labeled 'Manuscript'.

If you would like to make changes to your financial disclosure, please include your updated statement in your cover letter. Guidelines for resubmitting your figure files are available below the reviewer comments at the end of this letter.

If applicable, we recommend that you deposit your laboratory protocols in protocols.io to enhance the reproducibility of your results. Protocols.io assigns your protocol its own identifier (DOI) so that it can be cited independently in the future. For instructions see: [https://journals.plos.org/plosone/s/submission-guidelines#loc-laboratory-protocols](https://can01.safelinks.protection.outlook.com/?url=https%3A%2F%2Fjournals.plos.org%2Fplosone%2Fs%2Fsubmission-guidelines%23loc-laboratory-protocols&data=05%7C01%7Csteven.habbous%40ontariohealth.ca%7Cbe54471f52754efd03f708dbd56e4e87%7C4ef96c5cd83f466ba478816a5bb4af62%7C0%7C0%7C638338442184762483%7CUnknown%7CTWFpbGZsb3d8eyJWIjoiMC4wLjAwMDAiLCJQIjoiV2luMzIiLCJBTiI6Ik1haWwiLCJXVCI6Mn0%3D%7C3000%7C%7C%7C&sdata=ehyk%2F1tWouQtGlZdol17ezNHeKOEWJ79jM3DtPRbU9s%3D&reserved=0). Additionally, PLOS ONE offers an option for publishing peer-reviewed Lab Protocol articles, which describe protocols hosted on protocols.io. Read more information on sharing protocols at [https://plos.org/protocols?utm_medium=editorial-email&utm_source=authorletters&utm_campaign=protocols](https://can01.safelinks.protection.outlook.com/?url=https%3A%2F%2Fplos.org%2Fprotocols%3Futm_medium%3Deditorial-email%26utm_source%3Dauthorletters%26utm_campaign%3Dprotocols&data=05%7C01%7Csteven.habbous%40ontariohealth.ca%7Cbe54471f52754efd03f708dbd56e4e87%7C4ef96c5cd83f466ba478816a5bb4af62%7C0%7C0%7C638338442184762483%7CUnknown%7CTWFpbGZsb3d8eyJWIjoiMC4wLjAwMDAiLCJQIjoiV2luMzIiLCJBTiI6Ik1haWwiLCJXVCI6Mn0%3D%7C3000%7C%7C%7C&sdata=o8C%2B%2FTlOrustKmTsK0hh0%2F87cIQSRqfBRrgyDbS%2BK%2Fg%3D&reserved=0).

We look forward to receiving your revised manuscript.

Kind regards,

Franck Katembo Sikakulya
Academic Editor
PLOS ONE
