# Supplementary material for: Scheduled and urgent inguinal hernia repair in Ontario, Canada between 2010 and 2022: Population-based cross sectional analysis of trends and outcomes
Source: PLoS One. 2023 Dec 22;18(12):e0296258. doi: 10.1371/journal.pone.0296258 (PMC10745156; doi:10.1371/journal.pone.0296258)
Supplement: Attachment — Submitted filename: Response to reviewers - hernia repair - PONE-D-23-25270_R2.docx [file pone.0296258.s004.docx]

**December 6, 2023**

**PONE-D-23-25270R2**

Dear Dr Sikakulva,

On behalf of the co-authors, we thank you and the reviewers for the review. We have corrected the typographical error noted by the reviewer and conduced additional quality checks to ensure this error does not exist elsewhere.

Thank you for your time and consideration

Sincerely,

Steven Habbous, PhD

Epidemiologist

Ontario Health (Strategic Analytics)

Email: [steven.habbous@ontariohealth.on.ca](mailto:steven.habbous@ontariohealth.on.ca)

**December 4, 2023**

**PONE-D-23-25270R2**

Scheduled and urgent inguinal hernia repair in Ontario, Canada between 2010 and 2022: population-based cross sectional analysis of trends and outcomes

PLOS ONE

Dear Dr. Habbous,

Thank you for submitting your manuscript to PLOS ONE. After careful consideration, we feel that it has merit but does not fully meet PLOS ONE’s publication criteria as it currently stands. Therefore, we invite you to submit a revised version of the manuscript that addresses the points raised during the review process.

We look forward to receiving your revised manuscript.

Kind regards,

Franck Katembo Sikakulya
Academic Editor
PLOS ONE

**Additional Editor Comments:**


Please make changes as suggested by the reviewer

Thank you

**Reviewers' comments:**

**REVIEWER #1:**

This paper is much improved, and I now have only a few minor issues:

**Comment #1:** Thank you for your revisions. However, there now appear to be TWO Table 3's! Please correct this, and ensure that all Tables in the text or correct

**Response:** Thank you for catching this! We renamed the second Table 3 to Table 4, corrected this in the title page meta-data, and checked all references in the main body to any figures or tables, including the supplement to ensure we haven’t missed any others.

Thank you again for your diligence in reviewing this manuscript.
